# The Imperfections of the English Dentists' Act

**Published:** 1881-07

**Authors:** W. A. N. Catlin

**Affiliations:** President.


AMERICAN JOURNAL

OF

DENTAL SCIENCE.

Vol. XV. THIRD SERIES—JULY, 1881. No. 3.

### ARTICLE I.

### *The Imperfections of the English Dentists' Act, with Suggestions for Protecting the Rights of Qualified Surgeons.*

BY W. A. N. CATLIN, F. R. C. S., PRESIDENT.

An Address Delivered before the Association of Surgeons Practising Dental Surgery.

*Gentlemen*:—When last I had the honour to address you from this chair upon the subject of medical politics, I predicted that during my year of office the Parliamentary Committee of this Association would probably have an anxious duty to perform, inasmuch as there were then no less than three Medical Amendment Bills before Parliament, and our Sub-Committee were instructed not to neglect any opportunity of obtaining an amendment or repeal of the Dentists' Act.

A General Election put an end to Parliamentary business, and the matter of medical reform has since that time been fully discussed in the leading medical journals. The result has been very much in favour of the principles we advocate.

Sufficient time has now elapsed to enable the whole medical profession to form a calm and deliberate opinion as to whether the new Dental Scheme does or does not trench seriously upon its established rights, and whether our colleges are not sacrificing the best interests of the profession by granting license to practice special branches of Medicine or Surgery to persons who are not sufficiently educated to entitle them to be placed on the Medical Register.

A new Medical Amendment Bill will shortly be introduced, and as the subject of dental politics has now become a *medical question*, it will perhaps be desirable for us to consider what changes will be required in the Medi cal Act of 1858 to make it work equitable for the benefit of the public, and for the general good of the profession at large.

The Medical Act, in its Preamble, declares one of its objects to be, " to enable the public to distinguish between qualified and unqualified practitioners;" and it does this by establishing a Register for all those legally qualified. A correct list is published annually, and the public and the Courts of Law have free access to it.

The duty of preparing and publishing the Medical Register, and of compiling a new Pharmacopœia, has been entrusted to the General Medical Council, who also undertake the supervision of the education and examination of Medical Students.

The Register and the British Pharmacopœia have been completed, and are as perfect as it is possible to make them. Both these works reflect great credit upon the General Council.

The improvements, which have been suggested by the Medical Council in the department of general and medical education, have been many and great. It has laboured to establish a conjoint examination for England, Scotland and Ireland, to take the place of nineteen Examining Corporations.

The scheme for England has been agreed upon, and only awaits the co-operation of Scotland and Ireland. I am informed that the Reform Committee of the British Medical Association have hitherto opposed this scheme, upon the ground that it was not framed upon the principle of direct representation. Is universal suffrage the best form of election for this purpose? I think it is not. Altogether the General Medical Council has not given universal satisfaction in the management of its educational department, but it does not possess compulsory powers to enforce its recommendation.

After I have called your attention to some of the arrangements in the proposed conjoint examination for England, we will consider whether the constitution of the General Medical Council is such as is best fitted to carry out the duties it has to perform.

All who know how difficult it must have been to reconcile conflicting interests sufficiently to insure co-operation between the various examining Boards in England, must acknowledge that a great task has been performed in obtaining the harmony of opinion which now prevails with regard to conjoint examination. I believe the General Medical Council have approved the scheme which has been agreed upon, and will use its influence to bring about the co-operation of Scotland and Ireland.

The various English Institutions remain pledged to each other for two years more, and we may hope by that time all difficulties will have been overcome. If not, perhaps the Legislature will make the best form of examination compulsory.

These are some of the objections to the conjoint scheme:

1st. The present proposal to hold three separate examinations—first, second, third and final, by turn, in three different places, viz., the College of Physicians, the College of Surgeons, and Apothecaries' Hall, seems to me to sacrifice dignity to courtesy. It would surely be better to hold all the examinations at one place, perhaps on neutral ground, or say at the London University, the College of Physicians, or the College of Surgeons. I mean no disrespect to the Worshipful Apothecaries by omitting to name their Hall, but really there is something in appearance and locality (when it is not good) which makes an unfavorable impression upon the public mind.

2nd. That the short time fixed for examining a Student in some subjects, does appear to be insufficient to bring his knowledge to a fair test, and the omission of Dental Surgery in the examination seems, at least, to my prejudiced mind, a great disadvantage to the public. I think every General Practitioner should learn and practice it.

3rdly. That the course of study required for such an important branch of Surgery as Midwifery (only three months) is too short, and cannot fail to be insufficient. From time immemorial Obstetric Science has been imperfectly taught, and some of our examining bodies have altogether ignored it.

We have all had to run some risk at our birth from the chance of an incompetent Accoucher; and who can say that many infants (who might have turned out to be great men) have not been sacrificed to the neglect of our Colleges to cultivate this particular art?

We will now return to the consideration of our former subject (the constitution of the General Medical Council,) and it is the one of all other features of Medical Reform which has caused the greatest disputation. It is composed of twenty-three Members, and a Chairman, who is elected by the Members—twenty-four in all. Six Members are nominated by the Crown.

Sixteen are appointed by various Universities and Colleges in England, Scotland, and Ireland, and two by the Apothecaries' Halls of England and Ireland.

The predominance of corporated influence in this case is very plain.

The six Members nominated by the Crown being the only independent Members in the ordinary sense of the word.

It is, however, a remarkable fact that the men who have been selected to represent the Universities and Colleges are, for the most part, too high-minded and too honourable to stoop to petty influence, and hence they have performed their public duties with unswerving faithfulness. No institution that wishes to hold "undue influence" would have sent such men to the Council as Sir James Paget, Dr. Pitman, Dr. Humphry, and many others equally honest and independent. We have certainly been unusually fortunate in the characters of those who have been nominated by coporated bodies.

Still, it might have been otherwise, and it must be admitted that the principle upon which the General Medical Council has hitherto been constituted (if its usefulness is to be greatly enlarged) is bad upon the face of it.

The General Practitioners form the *bulk* of the profession, and they have large interests; but they are not adequately represented.

It is still a vexed question whether the Members of the General Medical Council should be all nominated by the Crown, whether all should be elected by direct representation, or whether the constitution of the Council should remain as it now is, with the addition of six Members to represent General Practitioners. I have carefully examined the evidence of first-rate men taken before a Committee of the House of Commons in 1879, and, as might have been expected, their opinions are most conflicting, and show strong symptoms of political bias.

The Report of the Executive Committee of the General Medical Council gives the written opinions of past and present Members of the Council, supplemented by memoranda by Dr. Pitman, Sir. James Paget, and Dr. Quain. These documents contain most interesting advice from persons who have been behind the scenes.

Although such men may fairly be supposed to be somewhat prejudiced, they have actual experience of the work to be done, and their evidence goes to prove that (so long as the duties are the same as now) election by direct representation would not supply the men best suited for the membership of the General Council.

On the other hand, the Reform Committee of the British Medical Association, a body of intelligent sound thinking men, who have given great attention to medical politics, are of opinion that the principle of direct representation should be brought into practical operation, not only for the election of members of the General Medical Council, but in every possible way. With this divergence of opinion, it is more than probable that the right course lies between the two extremes. Taking into account the high probability that additional duties of great importance will be added to the work already in the hands of the General Medical Council, it is of the utmost consequence that an agreement as to the future constitution of the Council should be arrived at by some sort of compromise before a Parliamentary Committee sits upon a new Medical Amendment Bill.

I have carefully studied the opinions of the opposing parties, and, under all the circumstances, I believe the direct representation, in some form or other, cannot any longer be resisted.

If the number of the Members of the Medical Council be allowed to remain the same as it now is, and six Members be taken from the Corporations and given to support the interests of General Practitioners, the Council might then be said fairly to represent all parties.

The six Members representing the whole body of the profession might be elected by direct representation every five years, each registered person having one vote. Frequent elections might be prevented by the whole Council having power to fill up any vacancy which might occur in the ranks of those representing the profession, until the time for a General Election arrived again.

It is evident, from the slow progress which has been made during the last twenty years, that the General Medical Council should be armed with compulsory powers to carry out every duty which may be entrusted to it. The Council should be looked upon as the backbone of any new Medical Act, and full powers must be given to it.

Time obliges me to leave the subject of Medical Reform, but I am convinced the Act of 1858 was well conceived and well intended by the Legislature. When it shall have been fortified by amendments, which experience has proved to be necessary to carry out its original intentions, and one or two clauses shall have been added to prevent piracy of professional rights, I believe we shall have before us a happier and a more prosperous future, and the profession will be more united and contented than it has been for some years past.

I have purposely abstained from alluding to the penal clauses of the Medical Act, because I think the Legislature will probably appoint some public prosecutor to enforce them by an easy and inexpensive process. It would be beneath the dignity of the Medical Council or any Society formed by the profession to undertake such work, and humiliating to a private individual, who would always be suspected of malice, whether he entertained such a feeling or not. On the whole, perhaps it would be better to leave the administration of the law entirely in the hands of the police. There could be no objection on the score of expense, as hitherto the amount of the penalties received has exceeded the cost of the prosecution.

I turn to the subject of dental politics with great reluctance. The dispute which is raging between the parties respecting the Dentists' Act has not been conducted in a satisfactory manner.

In 1859, a charter was obtained by the Royal College of Surgeons of England, which enables it to grant licenses in Dental Surgery to candidates who were little better than half educated up to the standard of medical fitness required from its Members.

Thus was permanently introduced the questionable principle of authorizing persons to practice an important branch of Surgery who had only obtained a limited knowledge of the science.

This bold step was undoubtedly an act of great injustice to every Fellow and Member of the College; yet they were in no way consulted in the matter.

The only precedent that I can find for granting a special degree in this country of late years is when the license in Midwifery was given by the English College of Surgeons to *three* persons who were not qualified practitioners, but the practice was immediately discontinued, and it has since been issued only to Members of the College.

The Dental License has been given to persons who are not Members of the College for twenty years, and the practice is still continued:

> "Tis strange such difference should be
> 'Twixt tweedle-dum and tweedle-dee."

The Dentists' Act goes further than the charter, and extends the same evil to the Scotch and Irish Colleges. It also encourages the mere "Dentist" to assume the prefix of "Surgeon," to the injury of those who have an exclusive right to the title.

Finally, by establishing a separate Register for Dentists (which the public may easily mistake for the Medical Register) it completely divorces that branch of Surgery from the whole science. These are the wrongs of which we, as Members of the College, complain.

The Dentists' Register is now crowded with ignorant pretenders, who must have made a false and fraudulent declaration, "that they were in practice as *bona fide* Dentists when the Act became law;" but as there is no legal definition of the words *bona fide* Dentist, I fear the General Medical Council will be unable to erase their names, and they will be a disgrace to the profession for a generation to come.

There will be a meeting of the Medical Council to-morrow (at a cost of about £400) to consider what can be done to remedy this condition of things, but, unless they enquire into each particular case, and act upon the rules of common sense, I see no way out of the dilemma.

The General Medical Council, under the circumstances, has power to expunge the names of impostors from the Dental Register, and their decision is final; but I fear they will be obliged to refer the matter to a court of law, and much time and money will probably be wasted before a legal decision can be obtained.

It has long been an established fact that a full knowledge of the science of medicine is absolutely necessary to the proper understanding and successful practice of any branch of Surgery, and on that ground the Legislature requires its public officers to hold the double qualification. By this it will appear that the licensing of half educated specialists is repugnant alike to the best interests of the public and to the medical profession at large. It also forms a bad precedent for future action.

My attention has been called by our Secretary to a short leader in the "Lancet," of October 30th, 1880. It goes so much to the point that I will ask your permission to quote it *in extenso.*

The editor says:

"It is interesting to notice the many proofs of ignorance or carelessness on the part of the public, in reference to the working of the 'Dentists' Act.'

"So far from that statute having any protective value for the community, it is, as we predicted it would and asserted it must be, a delusion and a snare. At present the Dentists' Register is a list of all persons, with or without knowledge and qualification, who were, or claimed to be, in the habit of tooth-drawing or making at the passing of the Act. Hereafter, that is to say when the present race of Dental Surgeons shall have died out, the Register will comprise the names of those imperfectly-educated and half-qualified persons who are simply Licentiates in Dentistry, and nothing more; together with such fully-qualified *Surgeons* as may elect to have their names classed with the professors of a 'specialty,' which, in so far as it is *surgical* cannot in the very nature of things have any *really* independent existence. We venture to think the number of medical men so minded will be exceeding small. Meanwhile the drawers of teeth and hewers of jaw-bones luxuriate in the possession of a Register, on which we regret to find the names of a few Surgeons. They also call themselves Surgeon-Dentists and Dental-Surgeons at pleasure, although there has been nothing surgical in their education, and they have no conceivable right to the title they claim. It is amusing to find members of the profession who, short-sightedly or perversely, agreed to accept the absurd phraseology of Sir John Lubbock's Bill, or forsooth thought it 'could do no harm,' now complaining of the results. They are simply the inevitable consequences of a serious misconception of the facts and a false policy. It would be ridiculous to pretend that we commiserate the Surgeons or greatly blame the Dentists who have taken advantage of their weakness; at the same time, we do pity the public who are victimized. The sooner qualified men take their names off the Dentists' Register, and open up some new mode of marking themselves off from the mass of specialists, the better it will be for the country at large and a branch of the medical profession which has been practically stultified."

A number of influential American Dentists have formed the Oral Society of Boston, and no one is allowed to become a member of it who is not a qualified Medical Practitioner. They also hold that all special degrees in medicine should be abolished.

This is a step in the right direction, and shows that the profession in the new country is becoming alive to the necessity of preventing its ranks from being overrun by a colony of mere specialties.

In recogniton of similar principles, which were initiated by this Association, the Oral Society of Boston have invited your Secretary and President to become Honorary

Members of it, to which they have assented, and I assure them we appreciate the compliment which has through you been paid to us.

It is simply idle to persuade any but the most prejudiced, that a partial education is sufficient for the successful practice of any branch of medicine. A greater deception was never fostered by the mind of man, and yet the chief promoters of the Dentists' Act have suffered themselves to be deluded by this false and ridiculous idea.

The surprising effects which may result from even slight pressure or irritation of a very small branch of our complicated nervous system (producing serious disturbance in remote parts,) can only be traced and understood by those who have a full and intelligent knowledge of its functions. The discoveries of Dr. Brown Sequard have shown how necessary this knowledge is to the practice of Dental Surgery. What self-educated Anatomist could trace the cause of deafness to irritation of the nerve of a tooth, unless he understood the great theory of reflex paralysis? And there are numerous complicated systems arising from diseased teeth (sometimes coupled with functional disturbance affecting the general health) which can only be skilfully treated by one who has a minute knowledge of all the resources of medical art.

It is natural that some persons, after they have obtained a full knowledge of their profession, attested by diploma, should prefer to give special attention to some branch for which they have a particular choice or aptitude; and to this there can be no sort of objection, but *special license is not required.* The public are quick to discover and avail themselves of superior abilities, if they be made known to them in the usual way by published works.

Having answered the fallacious argument that the education of a Surgeon is not necessary for a Dentist, I will ask and answer the question, What difficulties are there to prevent a Surgeon becoming a Dentist if he makes a special study of Dental Surgery *after he has taken his Diploma?*

I sincerely hope this practice will be adopted by young Surgeons.

It has been asserted that the time required to learn both Dentistry and Surgery would be too long, and that the hand can only be tutored in early youth to perform the delicate operations of Dental Surgery. This (so far as it is true) applies with equal force to delicate operations on the eye and other parts of the body, and yet there is no lack of skilful operators in every branch of Surgery.

Gold stopping is perhaps the only Dental operation which requires considerable practice, but the necessity for the use of that particular metal has been greatly exaggerated.

It is a fact, that has been proved by experiment and is confirmed by experience, that only the few (however well taught) acquire the power of manipulation which is necessary to make a *perfect* gold filling in awkward position within the mouth. Many large cavities in the molar teeth (if sufficient care be taken to prepare them) may be well filled by a person of ordinary skill with palladium amalgam, and some of the osteoplastic preparations are suited to the same operation in incisor and other front teeth. I do not desire to detract from the value of *perfect* gold stoppings, but I *do* say that only a few of the best Dentists can make them *perfect.* It is trifling with common sense to deny that all the other operations in Dental Surgery can be well performed by the Surgeon after a moderate term of experience. If further proof be required to contradict the false statement that proficiency in Dentistry can only be acquired in early youth, let the honorable and brilliant career of such men as Bell, Craigie, Harrison, Arnold, Rogers, and numerous others now living and equally skilful, who began to practice the specialty at mature age, proclaim the fallacy of such a doctrine. The truth is that mechanical talent is not uncommon, most men possess it, but in *different degrees.*

Moreover, a delicate sense of touch (as contra-distinguished from what is called a heavy-handed person) belongs to the individual, and is generally born with him. It is a perfection of touch which is the gift of nature, and art cannot imitate it. On the other hand, a person who is peculiarly rough may improve in manipulation by experience, but he will never attain to the same *degree* of perfection as that to which I have alluded as the gift of nature.

Purposely omitting any reference to the mechanical branch of Dentistry (*which I think should be practised separately,*) let us seriously enquire what there is in the Surgical branch which gives the slightest excuse for a special license. Verily I believe the manner in which this document has been forced upon the public is as unjust as it is impolitic. It is a monstrous fact (that no longer should be tolerated) that before the Student is allowed to pursue his studies at the Dental Hospital of London, he is required to give a written undertaking that it is his intention to take out the Dental license.

Having arrived at this point, let us consider what the public duty of this Association is under the vexatious difficulties that lie before it. I think we should endeavor to convince our opponents that we hald our opinions solely because we believe them to be right. I am not one of those who think that an intelligent minority should, either from the love of popularity or the dread of defeat, go with the multitude to do evil; but it is always wise to show polite deference for the opinions of others.

We should endeavour to obtain a just and reasonable compromise by the amendment of the Dentists' Act, to the extent which I have indicated, namely, that no one but a qualified practitioner should be allowed in future to receive the Dental license, or use the title of "Surgeon" alone or in conjunction with any other word or words, and that a clause to the same effect should also be inserted in the new Medical Amendment Bill :—that the Dentists' Register should be purged and be made CONSPICUOUSLY DENTAL :—and that those who have been tempted to take the Dental license, thinking it to be a more honourable degree than it really is, should have the way made easy for them to acquire the diploma of Member. I think this Association should show good feeling by helping them in every way. It is given to but few to possess so fine a judgment as will enable them to perceive and correctly estimate the course of coming events in great and complicated matters, and to fewer still amid the din of strife and the force of rivalry so to direct their conduct that it shall tend only to the public good. "Bad as the world is, respect is always paid to virtue." Even our bitterest enemies will not dare to raise the treacherous voice of slander against us if we oppose them with generosity, and ever keep in mind the good old maxim, "To err is human, to forgive divine."

I have already complained of the tone which has been adopted by those who are said to be the triumphant majority of our class. Even its talented leader has committed the mistake of using too great freedom of speech; when he compared this Association (consisting as it does of fully-qualified Surgeons) to a Society of "hysterical, timid ladies," I think he did not well use the great judgment he is said to possess. Supposing him to hold the opinion of Sir James Baker, "That despotic Government is a good thing, provided *he* is the despot, still common courtesy is due to gentlemen of his own rank in the same profession, although they may dare to differ with him in opinion.

I will not retort by recrimination, but recommend to his particular study the charitable lines of Burns—

"Then gently scan your brother man,  
Still gentler sister woman;  
Though they may go a kennen wrang—  
To step aside is human.  
One point must still be greatly dark,  
The moving *why* they do it;  
And just as lamely can ye mark,  
How far perhaps they rue it."

